# Object location and object recognition memory impairments, motivation deficits and depression in a model of Gulf War illness

**DOI:** 10.3389/fnbeh.2014.00078

**Published:** 2014-03-13

**Authors:** Bharathi Hattiangady, Vikas Mishra, Maheedhar Kodali, Bing Shuai, Xiolan Rao, Ashok K. Shetty

**Affiliations:** ^1^Research Service, Olin E. Teague Veterans' Medical Center, Central Texas Veterans Health Care SystemTemple, TX, USA; ^2^Institute for Regenerative Medicine, Texas A&M Health Science Center College of Medicine at Scott and WhiteTemple, TX, USA; ^3^Department of Molecular and Cellular Medicine, Texas A&M Health Science Center College of MedicineCollege Station, TX, USA

**Keywords:** depression, Gulf War illness, hippocampus dysfunction, novelty suppressed feeding test, open field test, object location test, object recognition test, voluntary exercise

## Abstract

Memory and mood deficits are the enduring brain-related symptoms in Gulf War illness (GWI). Both animal model and epidemiological investigations have indicated that these impairments in a majority of GW veterans are linked to exposures to chemicals such as pyridostigmine bromide (PB, an antinerve gas drug), permethrin (PM, an insecticide) and DEET (a mosquito repellant) encountered during the Persian Gulf War-1. Our previous study in a rat model has shown that combined exposures to low doses of GWI-related (GWIR) chemicals PB, PM, and DEET with or without 5-min of restraint stress (a mild stress paradigm) causes hippocampus-dependent spatial memory dysfunction in a water maze test (WMT) and increased depressive-like behavior in a forced swim test (FST). In this study, using a larger cohort of rats exposed to GWIR-chemicals and stress, we investigated whether the memory deficiency identified earlier in a WMT is reproducible with an alternative and stress free hippocampus-dependent memory test such as the object location test (OLT). We also ascertained the possible co-existence of hippocampus-independent memory dysfunction using a novel object recognition test (NORT), and alterations in mood function with additional tests for motivation and depression. Our results provide new evidence that exposure to low doses of GWIR-chemicals and mild stress for 4 weeks causes deficits in hippocampus-dependent object location memory and perirhinal cortex-dependent novel object recognition memory. An open field test performed prior to other behavioral analyses revealed that memory impairments were not associated with increased anxiety or deficits in general motor ability. However, behavioral tests for mood function such as a voluntary physical exercise paradigm and a novelty suppressed feeding test (NSFT) demonstrated decreased motivation levels and depression. Thus, exposure to GWIR-chemicals and stress causes both hippocampus-dependent and hippocampus-independent memory impairments as well as mood dysfunction in a rat model.

## Introduction

Gulf War illness (GWI) afflicts ~30% of the 700,000 military personnel who served in the Persian Gulf War-1 (PGW-1). Central nervous system (CNS) impairments are the most ubiquitous among the various symptoms of GWI (Amato et al., 1997; Haley et al., 2000a,b; Golomb, 2008; Parihar et al., 2013). These mainly comprise memory dysfunction, depression, concentration problems and insomnia (Haley et al., 2000a,b; Steele, 2000; Odegard et al., 2013). Although the exact causes of GWI are yet to be ascertained, it is widely believed that these enduring clinical symptoms are linked to a combination of exposures encountered by the service personnel during the PGW-1. These include significant exposures to the antinerve gas drug pyridostigmine bromide (PB), pesticides such as permethrin (PM, an insecticide), and N, N-diethyl-m-toluamide (DEET, an insect repellant) and war related stress. Exposure to PB occurred because veterans who were stationed in the battlefield areas (i.e., stationed within a mile of an exploding SCUD missile) believed to have consumed pyridostigmine bromide (PB) pills daily during the war for variable periods, as an antidote against the potential exposure to chemical weapons such as organophosphate nerve agents (Steele et al., 2012). Exposure to pesticides such as PM and DEET occurred because preparations for the PGW-1 comprised measures to offset the threat associated with infectious diseases transmitted via insects and ticks such as the use of pesticides for the area protection and uniforms and application of insect repellants on the skin.

Thus, veterans who served in the PGW-1 were significantly exposed to pesticide sprays and fogs used to kill flying insects, and insect repellants (Haley and Kurt, 1997; van Haaren et al., 2001; Institute of Medicine, 2010) in the period of their PB intake and war. Furthermore, a report by the research advisory committee (RAC) on GWI implies that the overall prevalence of GWI is greater in veterans who used higher amounts of pesticides than veterans who had limited exposure to pesticides during the PGW-1 (Binns et al., 2008). In some veterans, higher level of exposure to pesticides was also associated with a higher consumption of PB pills (Schumm et al., 2002; Abdullah et al., 2011). Other hypothesized causes of GWI include exposure to chemical weapons (for those veterans who were stationed near the chemical weapon depot demolitions), depleted uranium and oil well fires etc. However, based on both animal model and epidemiological studies, it is widely believed that GWI in a significant fraction of PGW-1 veterans is a consequence of synergistic interaction of PB with pesticides such as PM and DEET (Friedman et al., 1996; Hyams et al., 1996; Everson et al., 1999; Binns et al., 2008; Abdullah et al., 2011, 2012, 2013; Torres-Altoro et al., 2011; Steele et al., 2012; Ojo et al., 2013).

Considering the above, analyses of the long-term effects of such combined exposures using animal models have received significant attention (Abdel-Rahman et al., 2002, 2004; Abdullah et al., 2011). Our recent study in a rat model has shown that combined exposure to low doses of GWI-related (GWIR) chemicals such as PB, PM, and DEET (for 4 weeks) with or without stress (5 min of restraint stress) is detrimental for hippocampus function (Parihar et al., 2013). Such exposures caused spatial memory dysfunction in a water maze test (WMT) and increased depressive-like behavior in a forced swim test (FST). Interestingly, these memory and mood deficits were linked with several pathological changes in the hippocampus, which comprise chronic inflammation typified by the presence of ED-1+ activated microglial cells and hypertrophy of astrocytes, persistently decreased neurogenesis (a substrate important for hippocampal-dependent cognitive and mood function) and some loss of neurons in hippocampal principal cell layers (Parihar et al., 2013). In the current study, by performing both object location test (OLT) and novel object recognition test (NORT), we examined whether a larger cohort of animals exposed to GWIR-chemicals would display deficits for both hippocampus-dependent memory (measured through an OLT) and hippocampus-independent or partially hippocampus-dependent memory function (measured through an NORT). Furthermore, we determined whether increased depressive-like behavior observed earlier with an FST would be detectable in alternative tests for mood function such as voluntary exercise paradigm involving housing of rats in cages fitted with running wheels and novelty suppressed feeding test (NSFT).

## Materials and methods

### Animals

Three-months old Sprague-Dawley rats were obtained from Harlan (Indianapolis, IN). Following their arrival, animals were housed for 2 weeks in an environmentally controlled room (~23°C) with a 12:12-h light–dark cycle, and were given food (commercial rat chow) and water *ad libitum*. Animals were then randomly assigned to either the naïve control group or the GWI group (*n* = 16–28 per group) receiving exposure to GWIR-chemicals PB, PM, and DEET and 5 min of restraint stress for 4 weeks (here after referred to as GWI-rats). A separate vehicle (VEH) group undergoing oral gavage, shaving and handling was not included in this study, as our earlier study showed that such VEH-treated rats do not differ from age-matched naive control rats in terms of behavior or hippocampal cytoarchitecture (Parihar et al., 2013). All experiments were carried out in accordance with the National Institutes of Health guidelines for care and use of animals and in accordance with the animal protocol approved by the animal care and use committee of the Central Texas Veterans Health Care System, Temple TX.

### Application of GWIR-chemicals and stress, and timing of behavioral tests

**Figure 1:**
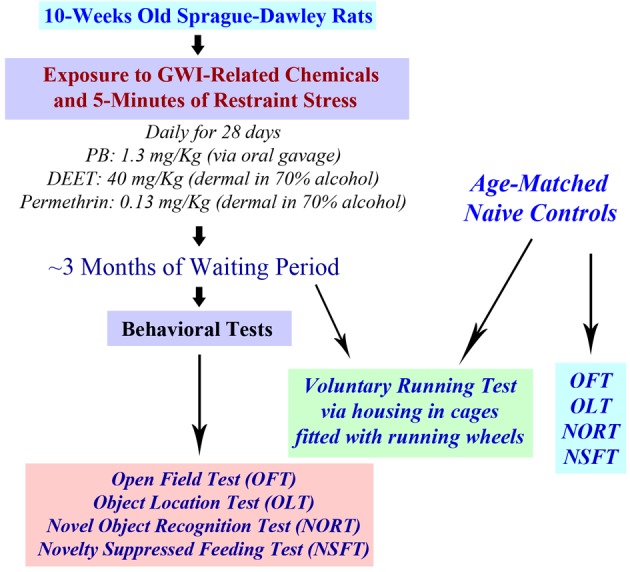
**A schematic showing the time-line of various experiments**. Rats were first exposed daily to Gulf War illness related (GWIR) chemicals and 5-min of restraint stress for 28 days. After a waiting period of ~3 months, a larger cohort of rats was subjected to behavioral tests in the following order: open field test (OFT), object location test (OLT), novel object recognition test (NORT), and novelty suppressed feeding test (NSFT). Another cohort of rats underwent a voluntary running test through housing in cages fitted with running wheels for 4 weeks. Cohorts of age-matched naive rats were also examined using all of these behavioral tests for comparison of data.

The chemical PB (1.3 mg/Kg; Sigma, St. Louis, MO) was administered through an oral gavage (500 μl in sterile water). Solutions of PM (200 μl, 0.13 mg/Kg in 70% alcohol; Chem. Service Inc., West Chester, PA) and DEET (200 μl containing 40 mg/Kg in 70% alcohol; Chem. Service Inc., West Chester, PA) and were applied sequentially to shaved skin areas located on the back of neck and between scapulae. A rat restrainer was used for the induction of 5 min of restraint stress, as described in our previous study (Parihar et al., 2011, 2013). The doses of PB, PM and DEET were chosen based on the previous studies of GWI using rats (Abdel-Rahman et al., 2002, 2004; Parihar et al., 2013). Behavioral tests were conducted 3 months after the exposure to GWIR-chemicals and stress. A time-line of experiments is illustrated in Figure 1.

### Analyses of anxiety-like behavior and general motor function using an open field test (OFT)

**Figure 2:**
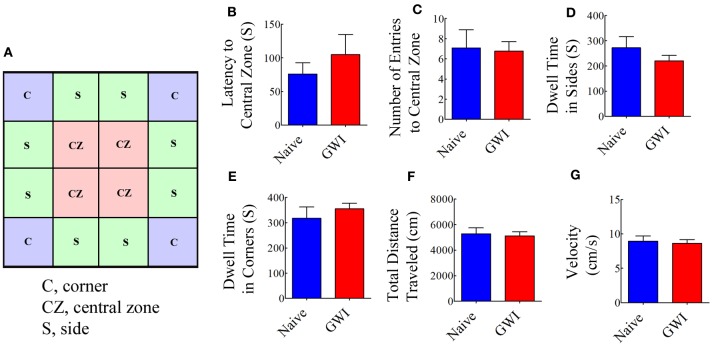
**Exposure to Gulf War illness related (GWIR) chemicals and stress does not result in increased anxiety or changes in general motor ability as revealed by an open field test**. A square box on the left **(A)** illustrates a schematic of the tested arena in an open field apparatus and shows areas that are designated as the central zone (CZ), sides (S), and corners (C). Bar charts compare latency values to first entry into the CZ **(B)**, number of entries into the CZ **(C)**, dwell time in sides **(D)**, dwell time in corners **(E)**, total distance traveled **(F)** and velocity of movement **(G)** between naive rats and GWI-rats at 3 months after the exposure. Two-tailed, unpaired Student's *t*-test revealed no differences between the two groups (*p* > 0.05). S, seconds.

We first examined both naive rats and rats treated with GWI chemicals and stress with an OFT to ascertain their relative anxiety levels and general motor ability. The testing arena (100 × 100 cm Plexiglas open field box) was divided into 16 squares (25 × 25 cm each) on the computer tracking system (Noldus-Ethovision). The resulting grid appeared on the computer screen as illustrated in Figure 2A. The four-squares located in the central area were defined as the central zone (CZ), the four squares located at each corner were designated as corners (C), and the remaining squares in the periphery were defined as sides (S). For assessing open field activity, each rat was placed in one of the corners of a brightly lit open field and its movement was video tracked for 10 min using Noldus ethovision XT system. The apparatus was cleaned with 70% alcohol and air-dried prior to the commencement of trial for every rat. The parameters measured comprised: (i) the latency to first entry into the central zone; (ii) numbers of entries into the central zone; (iii) dwell time in sides; (iv) dwell time in corners; (v) the total distance traveled; and (vi) mean velocity.

### Investigation of place recognition memory function using an object location test (OLT)

**Figure 3:**
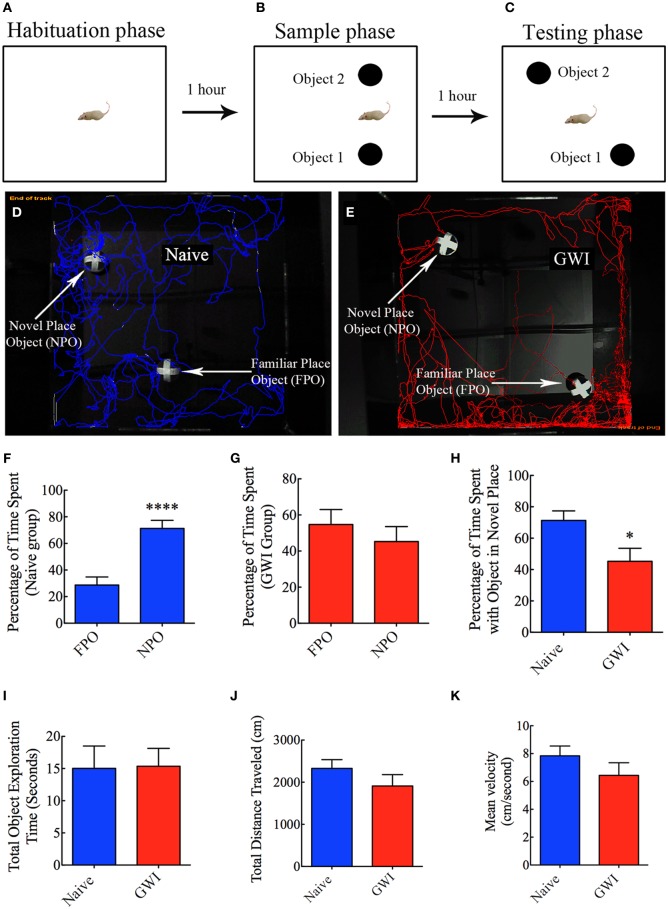
**Exposure to Gulf War illness related (GWIR) chemicals and stress causes location memory deficits as revealed by an object location test. (A–C)** Show a schematic representation of three trials and inter-trial intervals in this test. **(D,E)** Show representative track plots of a naive rat **(D)** and a GWI-rat **(E)** recorded by Noldus Ethovision XT tracking system in the testing phase. Arrows denote an object that was moved to a novel location (novel place object, NPO) and an object that remained in its original location (familiar place object, FPO) in the testing phase. Note that the naive rat spent more time exploring the moved object (see tracking lines around NPO and FPO in **D**) whereas the rat with GWI did not show preference to the moved object (see tracking lines around NPO and FPO in **E**). Bar charts in **(F–G)** compare percentages of time spent between the familiar place object (FPO) and the novel place object (NPO) in naive rats **(F)** and GWI-rats **(G)**. **(H–K)** Compare percentages of time spent with the object in the novel place **(H)**, the total object exploration time **(I)**, the total distance traveled **(J)**, and the velocity of movement **(K)** between naive rats and GWI-rats. Note that the general motor activity is similar between the two groups of rats **(J,K)**. ^*^p < 0.05; ^****^p < 0.0001 (two tailed, unpaired Student's *t*-test).

This test comprised three successive trials with an inter-trial interval of 60 min. The first trial comprised placing the rat in the center of an empty open field box [100 cm (L) × 100 cm (W) × 60 cm (H)] and allowing the rat to freely explore the box for 5 min (i.e., the habituation phase, Figure 3A). The second trial commenced 60 min after the first trial and involved placing the rat in the center of the same open field box having two identical objects on opposite sides of the box and allowing the rat to freely explore the objects for 5 min (i.e., the sample phase, Figure 3B). Following an inter-trial interval of 60 min, third trial was performed for 5 min through placement of the rat in the center of the same open field box with one of the objects remaining in the same location as in trial 2 and the second object moved to a new location in the open field box (i.e., the testing phase of OLT, Figure 3C). A rat is considered to be exploring an object when its nose is within 2 cm of the object. The movement of rat in the third trial (test phase) was continuously tracked and video recorded using Noldus Ethovision XT program. The apparatus was cleaned with 70% alcohol and air-dried prior to the commencement of each trial for every rat. Data such as times spent in exploring the object moved to a novel place, the object remaining in the familiar place, and total time spent in the object exploration were measured. Furthermore, the place discrimination index was calculated by using the formula, the time spent with the object moved to a novel place/the total time spent in exploring both the object moved to a novel place and the object remaining in the familiar place × 100. Then, the percentages of object exploration time spent with the object moved to a novel place vis-à-vis the object remaining in the familiar place were compared within each group. The novel place discrimination index was also directly compared between naïve rats and GWI-rats. Additionally, the velocity and the total distance moved during the third trial (test phase) was examined and compared between the two groups to ascertain whether depression (or lack of motivation) interfered with the place recognition memory testing. The preference of the rat to explore the object that has been moved to a new location reflects its ability for place recognition memory.

### Analyses of object recognition memory function using novel object recognition test (NORT)

**Figure 4:**
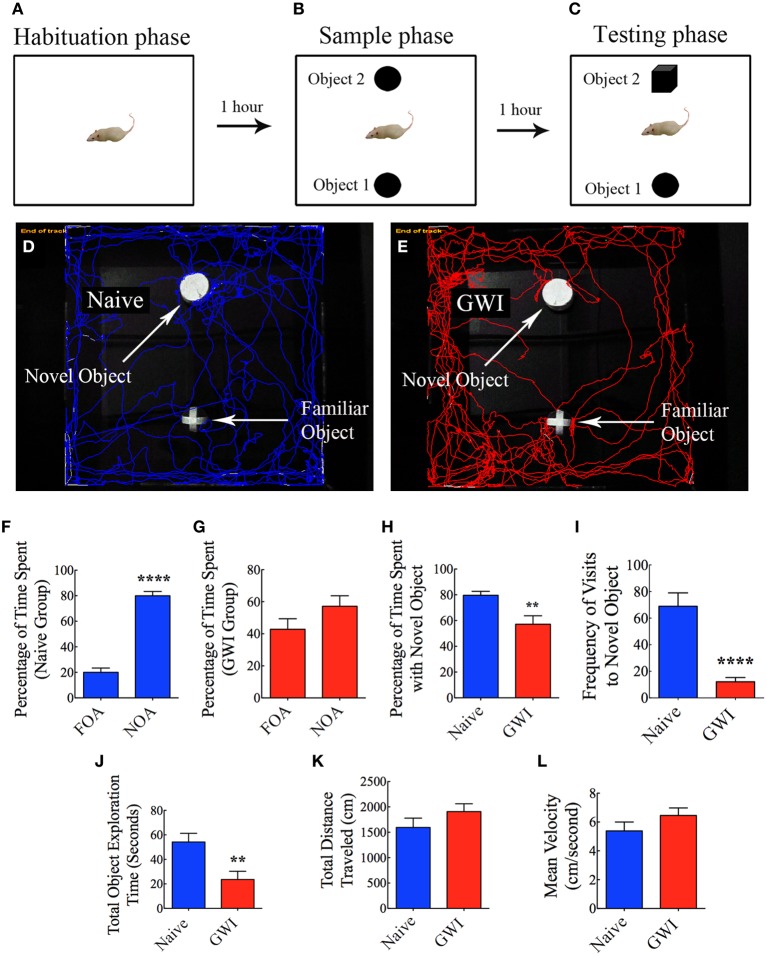
**Exposure to Gulf War illness related (GWIR) chemicals and stress causes object recognition memory deficits as revealed by a novel object recognition test. (A–C)** Show a schematic representation of three trials and inter-trial intervals in this test. **(D,E)** Show representative track plots of a naive rat **(D)** and a rat with GWI **(E)** recorded by Noldus Ethovision XT tracking system in the testing phase. Arrows denote locations of novel and familiar objects in the testing phase. Note that the naive rat spent more time exploring the novel object **(D)** whereas the rat with GWI did not show preference to the novel object **(E)**. Bar charts in **(F,G)** compare percentages of time spent between the familiar object area (FOA) and the novel object area (NOA) in naive rats **(F)** and GWI-rats **(G)**. **(H–L)** Compare percentages of time spent with the novel object **(H)**, frequency of visits to the novel object **(I)**, the total object exploration time **(J)**, the total distance traveled **(K)**, and the velocity of movement **(L)** between naive rats and GWI-rats. Note that the general motor activity is similar between the two groups of rats **(K,L)**. ^**^p < 0.01; ^****^p < 0.0001 (two tailed, unpaired Student's *t*-test).

This test also involved three successive trials for each rat with an inter-trial interval of 60 min (Hattiangady and Shetty, 2012). As described for OLT above, the first two trials comprised placing the rat in the center of an empty open field box and allowing the rat to freely explore the empty box for 5 min (first trial, the habituation phase, Figure 4A), and placing the rat in the center of the open field box having two identical objects on opposite sides of the box and allowing the rat to freely explore the objects for 5 min (second trial, the sample phase, Figure 4B). The third trial (the objection recognition memory testing phase) commenced 60 min after the second trial where the rat was allowed to explore objects for 5 min in the same open field box comprising one object used in the trial 2 (i.e., the familiar object) and a new object replacing the second object used in trial 2 (i.e., the novel object, Figure 4C). A rat is considered to be exploring an object when its nose is within 2 cm of the object. The movement of rat in the third trial was continuously tracked and video recorded using Noldus Ethovision XT program. The apparatus was cleaned with 70% alcohol and air-dried prior to the commencement of each trial for every rat. Data such as times spent in exploring the novel object, the familiar object and both objects (i.e., the total object exploration time) were collected. Furthermore, novel object discrimination index was calculated by using the formula, the time spent with the novel object/the total object exploration time ×100. Following this, the percentages of object exploration time spent with the novel object vis-à-vis the familiar object were compared within each group. The novel object discrimination index was also directly compared between naïve rats and GWI-rats. The velocity and the total distance moved during the third trial (test phase) was also collected and compared between the two groups to determine whether depression (or lack of motivation) interfered with the object recognition memory testing. The choice to explore the novel object more than the familiar object reflects the use of learning and (recognition) memory processes.

### Analyses of motivation level through measurement of voluntary exercise activity

To examine whether exposure to GWIR-chemicals and stress would impair the overall motivation of rats under normal conditions to voluntarily run in a rat activity wheel chamber, we individually housed both naïve rats and GWI-rats in larger cages fitted with rat running wheel. Such housing was provided for 12 h per day (during the dark cycle) for 4 continuous weeks. The equipment comprised a freely running activity wheel incorporated into the home cage and connected to a wheel counter having digital display to show the distance run by the rat (Lafayette instruments, IN, USA). Food and water were provided *ad libitum* inside the cage and the running activity of each rat was recorded every morning. Both groups of rats ran voluntarily for a period of 4 weeks and distances traveled per week were compared between the two groups.

### Characterization of depressive-like behavior using novelty suppressed feeding test (NSFT)

This test provides a sensitive and reliable measure of depression and motivation level in animals that resemble those in humans (Merali et al., 2003). In this test, both naïve rats and GWI-rats were first subjected to fasting for 24 h (by withdrawing food pellets from the cage) but were allowed to drink water during the fasting period. Following this, a single trial test was conducted in an open field Plexiglas chamber (100 × 100 × 60 cm) illuminated from above. Food pellets were placed in the middle of open field box in a shallow plastic container. Each rat was released from one of the corners and allowed to explore the open field box for 5 min and the movement of rat was video tracked using Noldus Ethovision XT program. The open field box was cleaned with 70% alcohol prior to testing and fresh food pellets were used for each rat to eliminate any odor related cues. Latency to the first bite of food was measured and compared between groups.

### Statistical analyses

For analyses of data in OFT, OLT, NORT, and NSFT, different parameters from the GWI group and the naive group were compared using a two-tailed, unpaired, Student's *t*-test. For the voluntary running test, a repeated measures Two-Way ANOVA with Bonferroni post-tests were employed for comparison of data between the two groups.

## Results

### Exposure to GWIR-chemicals and stress did not result in increased anxiety-like behavior or impair general motor ability in OFT

Open field test examined the conflict between the innate fear that rats have of the central area of a novel/brightly lit open field vis-à-vis their desire to explore the new environment. When anxious, the natural tendency of rats is to prefer staying closer to the walls or corners. Anxiety-related behavior was measured by the degree to which the rat avoided entering the central zone of the open field. Comparison of multiple parameters (such as the latency to the first entry into central zone, number of entries into the central zone; dwell time in sides and corners) demonstrated that GWI-rats did not exhibit increased anxiety-like behavior, in comparison to naive control rats (Figures 2B–E). Furthermore, parameters such as the total distance traveled and the velocity of movement were comparable between naive control rats and GWI-rats (Figures 2F,G), revealing that exposure to GWIR-chemicals and stress did not impair general motor ability of rats.

### Exposure to GWIR-chemicals and stress impaired hippocampus-dependent place recognition memory

In the OLT, naïve rats displayed a clear preference for the object moved to a novel place in comparison to the object that remained in the same (familiar) place, as these rats spent 71% of their object exploration time in the test phase with the object that was moved to a novel place (*p* < 0.0001, Figures 3D,F). In contrast, GWI-rats showed no preference for the object moved to a novel place, as they exhibited equal tendency to explore the object moved to a novel place (45% of the total object exploration time) and the object remained in the same place (55% of the total object exploration time, *p* > 0.05, Figures 3E,G). Representative track plots for a naive rat and a rat treated with GWIR-chemicals and stress show their relative activity around familiar place object and novel place object areas (Figures 3D,E). A direct comparison of the place discrimination index between the two groups clearly revealed the loss of place recognition memory function in GWI-rats (Figure 3H). However, total times spent in object exploration during the testing phase were comparable between the two groups (*p* > 0.05, Figure 3I). Moreover, total distances traveled and velocities of movement in the test phase were comparable between the two groups (*p* > 0.05, Figures 3J,K), consistent with the normal motor ability observed in GWI-rats in the OFT described above. These observations also implied that any underlying depression did not interfere with the place recognition memory testing in GWI-rats. Thus, exposure to GWIR-chemicals and stress clearly impairs hippocampus-dependent place recognition memory as measured by OLT.

### Exposure to GWIR-chemicals and stress resulted in novel object recognition memory dysfunction

In NORT, naïve rats displayed a clear propensity for exploring the novel object in comparison to the familiar object, as these rats spent 80% of their object exploration time in the test phase with the novel object (*p* < 0.0001, Figures 4D,F). On the other hand, GWI-rats showed no preference for the novel object, as their total object exploration time was more or less proportionately split between the novel object (57%) and the familiar object (43%, *p* > 0.05, Figures 4E,G). Representative track plots for a naive rat and a GWI-rat show their relative activity around familiar object and novel object areas (Figures 4D,E). A direct comparison of novel object discrimination index between the two groups clearly revealed the loss of object recognition memory function in GWI-rats (Figure 4H). Additionally, naïve rats visited the novel object more frequently than GWI-rats (*p* < 0.0001, Figure 4I). Although the total times spent in object exploration during the test phase were greater in naïve rats (*p* < 0.01, Figure 4J), total distances traveled and velocities of movement in the test phase were comparable between the two groups (*p* > 0.05, Figures 4K,L). These observations imply that any underlying depression likely did not interfere with the novel object recognition memory testing in GWI-rats. Thus, exposure to GWIR-chemicals and stress impairs novel object recognition memory function.

### Exposure to GWIR-chemicals and stress impaired motivation for voluntary running

**Figure 5:**
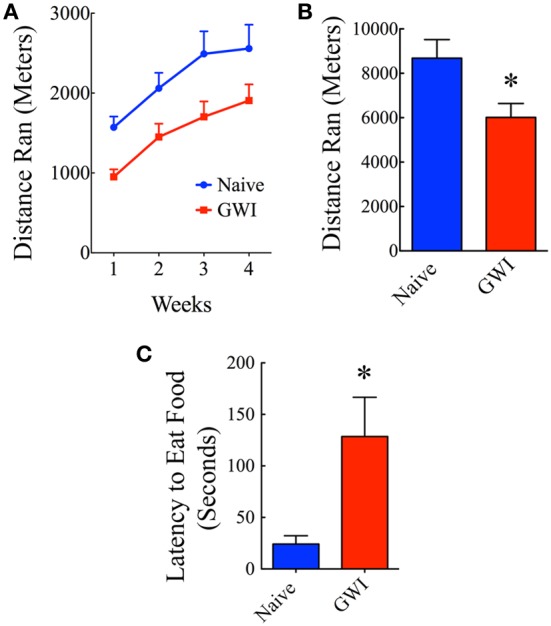
**Exposure to Gulf War illness related (GWIR) chemicals and stress causes alterations in motivation levels and depression. (A)** Compares distances ran by rats in the naive group and the GWI group over 4 weeks following housing in cages fitted with running wheels. Repeated measures Two-Way ANOVA revealed that animals in the GWI group displayed an overall reduced motivation for running than the naive control group (*p* < 0.05, *F* = 6.5). Bonferroni post-tests revealed that differences were significant in the 3rd week of running between the two groups (*p* < 0.05). However, both groups exhibited an increased motivation for running over the course of 4 weeks (*p* < 0.0001, *F* = 33.5). **(B)** Compares cumulative distances ran by the two groups in 4 weeks. **(C)** Compares latencies to eat food between the two groups in a novelty suppressed feeding test performed after 24 h of fasting. Note that latencies to eat food following fasting are greater in GWI-rats, implying depression in these animals. ^*^p < 0.05 (two tailed, unpaired Student's *t*-test).

Individual housing of rats in cages fitted with running wheels for 4 weeks revealed differences in motivation for voluntary running between naive rats and GWI-rats. Naïve rats ran ~1572 m during the first week, which was increased to 2060, 2492, and 2558 m in the second, third and fourth week respectively. Rats in GWI group ran ~952 m in the first week followed by 1451, 1703, and 1907 m in the second, third, and fourth week. The average cumulative distances ran by naive rats and GWI-rats were 8682 m and 6013 m respectively. Repeated measures Two-Way ANOVA revealed that GWI-rats displayed an overall reduced motivation for running than the naive control group (*p* < 0.05, *F* = 6.5, Figure 5A). Bonferroni post-tests revealed that differences were significant in the 3rd week of running between the two groups (*p* < 0.05). There was no interaction between treatment and time (p>0.05). Both groups exhibited an increased motivation for running over the course of 4 weeks (*p* < 0.0001, *F* = 33.5). Comparison of distances ran for the entire 4-week of running period demonstrated significantly reduced running activity in GWI-rats (*p* < 0.05, Figure 5B). Thus, GWI-rats showed significantly reduced motivation for voluntary exercise.

### Exposure to GWIR-chemicals and stress caused increased depressive-like behavior

In the absence of depression, rats subjected to fasting for 24 h move quickly toward the food pellets and start eating in NSFT. In contrast, rats with depression either take much longer time to reach and eat food or lack motivation to move toward the food. Thus, latency to the first bite of food served as a measure of the extent of depression in this test. Rats in GWI-group took significantly greater amount of time to eat food in comparison to naïve rats (*p* < 0.01, Figure 5C), which implied that they have an increased depressive-like behavior. This result is consistent with our earlier finding obtained through a FST (Parihar et al., 2013).

## Discussion

This study shows for the first time that exposure to low doses of GWIR-chemicals and 5 min of restraint stress for 4 weeks leads to deficits in object location memory and object recognition memory in a rat model. Additional studies revealed that these memory impairments were associated with decreased motivation levels for voluntary physical exercise as well as depression but not anxiety or deficits in general motor ability.

Our earlier study has shown that similar exposure to low doses of GWIR-chemicals and stress results in hippocampus-dependent spatial memory dysfunction in rats when measured through a WMT (Parihar et al., 2013). As WMT causes some amount of stress to rats (as a consequence of 28 swimming trials over a period of 7 days), we pondered whether memory deficits observed in WMT be reproducible with other hippocampus-dependent cognitive tests that are considered relatively stress free. To resolve this, we examined a larger cohort of animals with OLT at 3 months after their exposure regimen in a stress-free environment in this study. Each rat first explored two identical objects in the acquisition phase of the OLT. An hour later, the rat explored the objects again with one of the objects moved to a new location. Naive rats spent more time in exploring the moved object than the object that remained in the same position as per expectation, which confirmed their ability for remembering which spatial locations have or have not been engaged earlier (Warburton et al., 2013). However, GWI-rats showed no preference for the moved object as they spent nearly equivalent amounts of time with the object in a novel place and the object in the familiar place, which confirmed that these rats have object location memory dysfunction. Previous lesion studies have demonstrated that positive functioning of this task critically requires the hippocampus but not the perirhinal cortex or the medial prefrontal cortex (Ennaceur et al., 1996; Bussey et al., 2000; Barker and Warburton, 2011; Warburton et al., 2013). This is consistent with the well-accepted notion that the hippocampus supports a cognitive map of the external world and that the hippocampus is part of a memory system that stores information about places in the organism's environment, their spatial relations, and the existence of specific objects in specific places (O'Keefe and Conway, 1978; Manns and Eichenbaum, 2009). From this perspective, the findings confirm that animals exposed to low doses of GWIR-related chemicals and stress indeed exhibit hippocampus-dependent memory dysfunction. This could be due to several alterations in the hippocampus that were discovered in our previous study such as greatly decreased neurogenesis in the dentate gyrus, inflammation in the form of hypertrophied astrocytes and activated microglial cells, and partial loss of principal neurons in the CA1 and CA3 subfields of the hippocampus (Parihar et al., 2013). This is because, adequate amounts of hippocampal neurogenesis is widely believed to be important for making new memories (van Praag et al., 2005; Deng et al., 2010; Parihar et al., 2011; Wang et al., 2012) and partial neurodegeneration and inflammation can interfere with hippocampus function (Krishnadas and Cavanagh, 2012; Kohman and Rhodes, 2013).

It is of great interest to note that the hippocampus dependent memory impairment observed in our rat model is consistent with the hippocampus dysfunction noted in veterans with GWI. Initially, a large number of veterans with GWI have self-reported cognitive deficits and memory problems. Following this, MR spectroscopy and single photon emission computed tomography (SPECT) analyses in veterans with GWI have suggested hippocampus dysfunction (Haley et al., 2000a,b, 2009; Menon et al., 2004). Furthermore, hippocampal dysfunction in veterans with GWI has been confirmed with measures of left and right hippocampal function assessed through arterial spin labeling, which involved intravenous infusions of physostigmine and measurement of the regional cerebral blood flow in hippocampi (Li et al., 2011). More importantly, a recent study using a face-name associative recall test (an objective measure of memory directly linked to hippocampus function) in association with measurements through functional magnetic resonance imaging (fMRI) demonstrated memory deficits in ill GW veterans (Odegard et al., 2013). In this study, GW veterans with or without illness completed a face-name memory paradigm for which they were to connect two distinct pieces of data such as faces and names (Odegard et al., 2013). They were shown the studied faces afterward and queried to ascertain if a face had been allied with a name at study and whether they could recall the name if it was associated with a particular face. The fMRI data showed that performance on the memory test was directly related to the amount of activation in the left hippocampus observed during study (Odegard et al., 2013).

To determine whether GWI-rats also exhibit memory impairments that are not dependent on the hippocampus, we examined these rats for object recognition memory function using another stress free test. For this, we employed an NORT comprising exploration of two identical objects in the acquisition phase and comparison of the exploration of a familiar and a novel object an hour later in the test phase. This test does not involve the hippocampus but critically requires the integrity of the perirhinal cortex (Bussey et al., 2000; Langston et al., 2010). Naive rats spent more time in exploring the novel object than the familiar object, which confirmed their ability for object recognition memory. However, GWI-rats showed no preference for the novel object as they spent nearly equivalent amounts of time with the novel and familiar objects, implying that these rats have object recognition memory dysfunction. These results are different from our previous study where GWI-rats demonstrated normal object recognition memory function when minimal inter-trial interval (5 min) was maintained between the acquisition and test phases. This suggests that GWI-rats display deficits for long-term object recognition memory function. This is likely due to alterations in the function of the perirhinal cortex (such as the expression of plasticity associated with recognition memory), as studies have shown that changes in the NMDA receptor neurotransmission in the perirhinal cortex can impair object recognition memory (Griffiths et al., 2008; Warburton et al., 2013). Hence, studies on changes in the structure and function of the perirhinal cortex will be needed in the future to recognize mechanisms underlying object recognition memory dysfunction in GWI-rats. While the human perirhinal cortex (Brodman areas 35 and 36) involved in both visual perception and memory (Murray et al., 2007) has not been specifically examined in veterans with GWI, a study suggests general cortical atrophy and baseline working memory compensation in the basal ganglia of a subset of veterans with GWI (Rayhan et al., 2013).

We also examined whether object location and object recognition memory deficits were associated with anxiety or changes in motivation levels for voluntary physical exercise and depression. Analyses of various parameters in a 10-min OFT did not show increased anxiety in GWI-rats. Furthermore, in both OLT and NORT, GWI-rats traveled distances and displayed velocities that are highly comparable to naive control animals. These results suggest that exposure to GWIR-chemicals did not induce anxiety. These OFT results are however different from our previous findings in a 5-min elevated plus maze test (EPMT) where animals treated with GWIR-chemicals and stress exhibited signs of anxiety in the form of reduced entries to open arms and reduced dwell time in open arms (Parihar et al., 2013). This discrepancy likely reflects differences in the duration of test (5 min in EPMT vs. 10 min in OFT) and the less complex environment encountered in an OFT. Thus, GWI-rats do not seem to exhibit a major anxiety disorder but show a tendency for increased fear when they encounter a complex environment such as in an EPMT. However, these animals clearly showed decreased motivation for leisure activity (a sign of depression), which was evidenced in this study through significantly reduced motivation for voluntary exercise using running wheels incorporated in their cages. Moreover, depression analyses using an NSFT clearly revealed increased depressive-like behavior in these rats, which is also consistent with our earlier findings using an FST (Parihar et al., 2013). Depression is likely also linked to some of the alterations observed in the hippocampus in our previous study such as greatly waned neurogenesis, inflammation, partial loss of principal neurons and reduced volume (Parihar et al., 2013). Particularly, reduced hippocampal neurogenesis may be having a major role in the pathophysiology of depression in this model, as multiple previous studies have showed evidence that mood function depends on the extent of hippocampal neurogenesis (Santarelli et al., 2003; Snyder et al., 2011; Eisch and Petrik, 2012; Kheirbek et al., 2012)

## Conclusions

The results of this study reinforce that exposure to GWIR-chemicals and stress causes both hippocampus-dependent and hippocampus-independent memory dysfunction as well as mood impairments. From this viewpoint, this animal model recapitulates the major CNS symptoms seen in veterans with GWI, as memory and mood deficits being the most common of symptoms (Odegard et al., 2013; Smith et al., 2013). Therefore, this is an ideal animal model for testing drugs that have promise for treating GWI. Considering the underlying hippocampal pathology identified in our earlier study (Parihar et al., 2013), future studies need to examine drugs that have promise for enhancing memory and mood function through increased neurogenesis and reducing inflammation in the hippocampus.

## Author contributions

Bharathi Hattiangady and Vikas Mishra contributed equally to this work. Bharathi Hattiangady contributed to the experimental design for the various behavioral studies, analyzed and interpreted data, prepared figures and wrote the first version of manuscript text. Vikas Mishra performed several behavioral experiments, analyzed and interpreted data, and contributed toward the preparation of figures. Maheedhar Kodali performed some of the behavioral studies, analyzed data and contributed to preparation of figures. Bing Shuai and Xiolan Rao performed chemical and stress exposures to animals and contributed to behavioral experiments and collection of data. Ashok K. Shetty conceived the study, conceptualized behavioral paradigms, interpreted data, and prepared the final version of manuscript text. All authors gave input to the manuscript text and approved the final version of the manuscript.

### Conflict of interest statement

The authors declare that the research was conducted in the absence of any commercial or financial relationships that could be construed as a potential conflict of interest.
